# Loss of Y and clonal hematopoiesis in blood—two sides of the same coin?

**DOI:** 10.1038/s41375-021-01456-2

**Published:** 2021-11-01

**Authors:** Viktor Ljungström, Jonas Mattisson, Jonatan Halvardson, Tatjana Pandzic, Hanna Davies, Edyta Rychlicka-Buniowska, Marcus Danielsson, Paul Lacaze, Lucia Cavelier, Jan P. Dumanski, Panagiotis Baliakas, Lars A. Forsberg

**Affiliations:** 1 Department of Immunology, Genetics and Pathology, Science for Life Laboratory, Uppsala University, Uppsala, Sweden; 2 Department of Clinical Genetics, Uppsala University Hospital, Uppsala, Sweden; 3 Faculty of Pharmacy and 3P Medicine Laboratory, International Research Agendas Programme, Medical University of Gdańsk, Gdańsk, Poland; 4 Public Health Genomics, Department of Epidemiology and Preventive Medicine, School of Public Health and Preventive Medicine, Monash University, Melbourne, VIC Australia; 5 The Beijer Laboratory, Uppsala University, Uppsala, Sweden

**Keywords:** Risk factors, Diseases, Immunology, Genetics research, Genetics

## To the Editor:

Studies in recent years have revealed that increasing age is associated with the accumulation of post-zygotic genetic aberrations in different cell lineages even in the absence of active malignancy. Clonal hematopoiesis of indeterminate potential (CHIP) is defined as the detection of somatic mutations in genes commonly associated with myeloid neoplasms in the peripheral blood of individuals with no sign of hematological malignancy. The process of CHIP derives from ageing hematopoietic stem cells that have accumulated mutations, rendering proliferative advantage compared with their peers, resulting in clonal expansion [1, 2]. CHIP is an age-related phenomenon, regularly observed in healthy older individuals at frequencies up to 10% at age 70 years. CHIP has been associated with increased risk of hematological malignancies as well as cardiovascular diseases [1–3].

In parallel, peripheral leukocytes often show mosaic loss of chromosome Y (LOY) in ageing men [4], detectable in more than 40% of the men above the age of 70 years, in the UK Biobank [5]. Mosaic LOY manifests as a fraction of an individual’s leukocytes lacking the Y chromosome. In longitudinal studies, LOY typically increases in frequency over time, comparably to the process of clonal hematopoiesis [6]. Remarkably, recent single-cell analyses of leukocytes from men diagnosed with Alzheimer’s disease (median age 80 years) identified leukocytes with LOY in every studied subject [7]. This has established LOY as the most common post-zygotic mutation in the hematopoietic lineages of aging men. Risk factors for LOY in leukocytes include age, smoking, and germline genetic predisposition [5, 8, 9]. Leukocytes with LOY in peripheral blood are associated with increased risk for all-cause mortality [4, 9], hematological and non-hematological cancers [4, 10, 11] and other age-related disorders such as Alzheimer’s disease, diabetes, and cardiovascular events [9, 12, 13].

Hence, carriers of leukocytes with post-zygotic mutations—including LOY and CHIP—display an increased risk for diseases both inside and outside of the hematopoietic system. The mechanisms behind these associations, however, remain to be established, as does the relative contribution of these abnormalities to disease etiology [14]. Recent studies suggest that LOY in leukocytes could confer direct physiological effects through LOY-associated transcriptional effects affecting global gene expression, and acting as a biomarker of genomic instability in somatic tissue [5, 7]. Considering the similarities in age-related prevalence and disease risks conferred by LOY and CHIP, it is of considerable interest to determine whether the two phenomena co-exist or might occur in a mutually exclusive manner [15]. Of note, a recent study revealed a co-occurrence of LOY and CHIP in bone-marrow cells derived from patients referred for clinical bone-marrow evaluation [11].

**Fig. 1:**
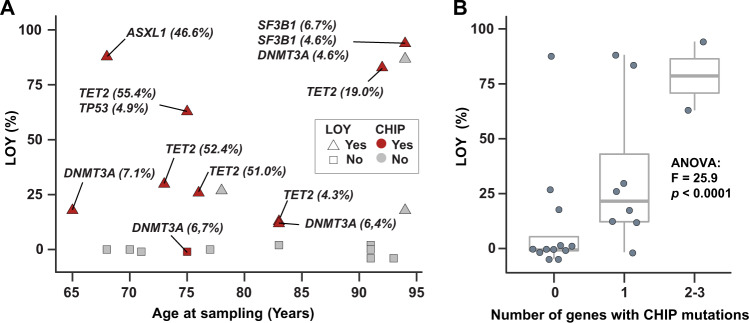
Co-occurrence of LOY and CHIP in monocytes of ageing men. **A** Shows the level of LOY (%) in each monocyte sample (*y*-axis) plotted against the age in years of the sampled men. The color (red/gray) of each dot indicates whether a pathogenic CHIP mutation was detected in the sample, while shape (triangle/circle) denotes if the sample was classified as having LOY or not. The gene names and variant allele frequency (VAF) of the CHIP mutations are labeled next to the sample in which CHIP was present. The boxplot in **B** displays the relationship between different levels of LOY (%, *y*-axis) and the number of genes in which CHIP was detected (*x*-axis).

To investigate whether LOY and CHIP may co-exist in peripheral blood of healthy individuals, we investigated the co-occurrence of LOY and CHIP in monocytes derived from 24 healthy men. Details on the studied cohort is provided in Supplementary Table 1. The men had no evidence of hematological disease, and the LOY-status in each sample had previously been established by SNP-array analyses of FACS isolated monocytes [7]. We sequenced monocyte-derived DNA collected from men with high or undetectable levels of LOY, using a TruSight sequencing panel targeting 54 genes often mutated in myeloid neoplasms (Supplementary Table 2). Two samples (one with LOY and one without) were excluded from final analysis after standard QC filtering of the sequencing data. The level of LOY mosaicism and CHIP mutations detected in the 22 age-matched samples are illustrated in Fig. 1 (e.g. 12 samples with LOY; median age = 83, range = 65–94 and 10 without evidence of LOY; median age = 83, range = 68–94). Pathogenic CHIP variants were detected in the following genes: *TET2* (*n* = 5), *DNMT3A* (*n* = 4), *SF3B1* (*n* = 2), *ASXL1* (*n* = 1), *TP53* (*n* = 1), with a median variant allele frequency (VAF) of 20.74% (range 4.3–55.4%). The gene panel also detected a set of CHIP variants classified as variants of uncertain significance, which consisted of: *BCOR* (*n* = 5), *ZRSR2* (*n* = 3), *BCORL1* (*n* = 1), *FBXW7* (*n* = 1), *FLT3* (*n* = 1), *GATA2* (*n* = 1), *KDM6A* (*n* = 1), *KIT* (*n* = 1). However, only known pathogenic variants were included in the final analysis and Fig. 1. Further details on identified CHIP variants are provided in Supplementary Table 3.

Pathogenic CHIP variants were detected in 10 of the 22 (45%) men with the striking observation that 9 of 12 (75%) of men with LOY also carried mutations in genes known to be associated with CHIP. This compared with CHIP being detected in only 1 of 10 men (10%) of men without LOY. A Fisher’s exact test demonstrated a significant overrepresentation of CHIP mutations in samples with LOY (*p* = 0.0037). Further ANOVA testing compared the number of genes with pathogenic CHIP mutations with the level of LOY, and showed larger number of genes were affected with CHIP mutations in samples with higher levels of LOY (*F* = 25.9, *p* < 0.0001, Fig. 1B). A closer investigation of the nine cases with simultaneous occurrence of LOY and CHIP suggested that the two types of post-zygotic genetic abnormalities often co-existed in the same or closely related hematopoietic lineages. Hence, in samples with evidence of LOY in ≥25% of the cells and detected CHIP mutations, 5/6 (83%) also displayed a high VAFs (mean 44.9%, range 19.0–55.4%). In contrast, in three samples with CHIP and LOY in <25% of cells, the range of VAFs were lower (4.3%, 6.4%, and 7.1%). These results suggest a frequent co-existence of LOY and CHIP in the studied monocytes, indicative of clonal expansion of hematopoietic stem cells carrying both lesions. However, we also observed cases with LOY without CHIP mutations, and vice versa (Fig. 1). Therefore, larger studies are needed to further describe and validate the inter-relationship between different types of post-zygotic mutations in monocytes, as well as other hematopoietic lineages.

The relationship between advanced age and cancer is well established. Findings of cancer-related aberrations in elderly individuals without known malignancy would therefore further corroborate the hypothesis that cancer stems from a step-wise accumulation of mutations leading to a malignant phenotype. CHIP and LOY are two such examples. Although they have previously been studied individually, the relationship between the two phenomena is not fully elucidated, but may have important clinical implications. Our results are in line with a recently published study, which revealed that a large proportion of cases with ≥75% LOY estimated by karyotyping also carried CHIP-associated mutations [11]. Interestingly, the latter analysis was performed on samples from patients referred for bone-marrow evaluation, indicating some evidence of one or more hematological lineages being affected in peripheral blood, skewing the material towards the overt pre-malignant or malignant spectrum. Our study serves as a validation that the association between LOY and CHIP is not restricted to the bone-marrow of patients with potential hematological disease. A limitation of our study is the targeted sequencing approach covering 54 recurrently mutated genes in MNs as well as the application of a 4% VAF cut-off, and a relatively small sample size. Our approach may have led to the underestimation of CHIP due to the higher VAF threshold used compared with previous studies, and the possibility of rare CHIP mutations being missed by targeted sequencing. However, the parameters for variant filtration used in our study closely resemble those used in a clinical setting, thus likely accurately reflecting the definition of CHIP used in clinical practice. Moreover, the most commonly reported CHIP genes are included in the panel that was used in our study.

Considering the growing list of diseases associated with leukocytes carrying post-zygotic LOY and CHIP mutations, it has become evident that pre-malignant lesions in immune cells are risk factors not only for development of subsequent hematological neoplasms, but also for other more common diseases in other organ systems. Understanding the complex and varied relationships that may exist between different classes of age-related somatic aberrations, as well as their effects on leukocyte function and clinical outcome, needs further evaluation [14, 15]. Our findings are the first, to our knowledge, to demonstrate a co-occurrence of LOY and pathogenic CHIP variants in individuals without any evidence of hematological disease. We also show that men with higher level of LOY mosaicism tend to have higher CHIP VAFs, which suggests that these lesions may co-exist within the same clone. Taken together, our results suggests that CHIP and LOY often occur concurrently in ageing bone-marrow, and therefore could be viewed as two sides of the same coin.

## Supplementary information

Supplementary information
