# Supplementary material for: Loss of Y and clonal hematopoiesis in blood—two sides of the same coin?
Source: Leukemia. 2021 Nov 1;36(3):889–91. doi: 10.1038/s41375-021-01456-2 (PMC8885420; doi:10.1038/s41375-021-01456-2)
Supplement: Supplementary file 1 — Supplementary information [file 41375_2021_1456_MOESM1_ESM.pdf]

Supplementary Information

Loss of Y and clonal hematopoiesis in blood - Two sides of the same coin?

Ljungström *et al.*

Content Page

Content	Page
Supplementary Methods	2-3
Supplementary Table 1	4
Supplementary Table 2	5
Supplementary Table 3	6

Samples and LOY analysis

We analyzed 24 samples of monocyte derived DNA with known LOY-status for evidence of CHIP mutations. The samples were selected from a collection included in a recently published paper (1) in which blood was collected from 408 male subjects in Uppsala, Sweden and Kraków, Poland. Thus, peripheral blood mononuclear cells was previously isolated and sorted into different cell types with FACS, including monocytes that were defined by side and forward scatter (SSC and FSC) and CD14+. Genotyping using Illumina SNP-arrays was performed and the level of LOY in each sample was estimated by calculation of the median Log R Ratio of probes located in the male specific part of chromosome Y (mLRRY) using the genotyping data, as described (1, 2). After correcting for batch effects with a local regression median, mLRRY values was transformed into percentage of cells with LOY in each sample using a validated method (i.e. % LOY = $100\cdot(2^{2\cdot mLRRY})$ (3). The samples included in the present analysis was collected from men with no evidence of hematological malignancies and selected based on LOY-status, age at sample collection and sample availability. In the selected sample set, 13 monocyte DNA samples displayed LOY while 11 did not. The research has been approved by local research ethics committee in Uppsala, Sweden (Regionala Etikprövningsnämnden in Uppsala (EPN): Dnr 2013/350; Dnr 2015/092; Dnr 2015/458; Dnr 2015/458/2, the latter with update from 2018) and the Bioethical Committee of the Regional Medical Chamber in Kraków, Poland (Komisja Bioetyczna, Okregowa Izba Lekarska, Kraków, Nr: 6/KBL/OIL/2014). All participants have given their written informed consent to participate.

DNA sequencing and bioinformatics analysis

Sequencing libraries were prepared from 50 ng of genomic DNA using the Trusight Myeloid Sequencing Panel (Illumina, San Diego, California) according to manufacturer's instructions. The panel consists of 568 unique amplicons covering either hotspots or full coding region of 54 genes frequently mutated in myeloid neoplasms. After PCR clean-up, double-stranded DNA quantity of individual libraries was assessed using Qubit dsDNA HS Assay kit and the fragment size distribution was determined with an Agilent 2200 Tapestation system (Agilent, Technologies) using the high sensitivity

D1000 Screen Tape. A pool of equimolar libraries was created and denatured. Paired-end sequencing was performed on an Illumina NextSeq instrument as specified by the manufacturer.

FASTQ-demultiplexing and adapter removal was performed using bcl2fastq (v2.20.0.422). The sequencing reads were aligned to the GRCh37 human reference genome using bwa-mem (v. 0.7.16) and post-processed using Samtools (v1.8). Pisces (v5.2.10.49) was used for detection of single nucleotide variants (SNVs) and small indels with non-stringent detection thresholds of 40x coverage and variant allele frequency (VAF) 0.5%. All variants were filtered against an in-house artefact blacklist compiled from previous sequencing of > 3000 samples with the same panel design and subsequently analyzed for highly recurrent variants indicative of run-private sequencing artefacts. Finally, all variants were stringently filtered with a VAF cutoff of 4%, read depth > 1000x, and minimum 100 reads supporting the variant allele. Variants were annotated with population variation databases and Cosmic (v85) using VEP (v91) and SnpEFF (v4.3). Variants had to meet the following conditions to be included in downstream analysis: (i) located within an exonic or splicing region; (ii) be non-synonymous; (iii) have a maximum allele frequency in 1000 genomes European and gnomAD non-Finnish European of 0.3%.

Statistical analyses

Hypothesis testing in the form of Fisher's exact test and ANOVA was performed in R (v3.6.3). Fisher's exact test was used for the association between LOY and CHIP amongst samples. A type III ANOVA was used to estimate the effect of CHIP on the percentage of LOY cells, with LOY level as a continuous response variable and the number of genes with CHIP variants as the explanatory factor. The type III ANOVA was run using the *car* package and with specified contrasts (*contr.sum* and *contr.poly*).

Supplementary Table 1. Summary of studied cohort.

	Group of subjects with detectable LOY in monocytes	Group of subjects withot detectable LOY in monocytes
**Number of subjects**	12	10
**Median age at sampling (range)**	80,0 (68-93)	80,5 (65-94)
**Number of current smokers**	1	1
**Number of previous smokers**	6	6
**Number of subjects with any hematological malignancy**	0	0
**Number of subjects with any non-hematological cancer**	0	0
**Number of subjects with any type of dementia**	0	0

Supplementary Table 2. Genes and exons included in the TruSight myeloid panel.

Gene	Region included	Gene	Region included
ABL1	exon 4-6	*KIT*	exon 2, 8-11, 13 and 17
ASXL1	exon 12	*KRAS*	exon 2-3
ATRX	exon 8-10 and 17-31	*MPL*	exon 10
BCOR	entire gene	*MYD88*	exon 3-5
BCORL1	entire gene	*NOTCH1*	exon 26-28, 34
BRAF	exon 15	*NPM1*	exon 12
CALR	exon 9	*NRAS*	exon 2-3
CBL	exon 8-9	*PDGFRA*	exon 12, 14 and 18
CBLB	exon 9-10	*PHF6*	entire gene
CBLC	exon 9-10	*PTEN*	exon 5 and 7
CSF3R	exon 14-17	*PTPN11*	exon 3 and 13
DNMT3A	entire gene	*RUNX1*	exon 2-9
ETV6	entire gene	*SETBP1*	5% of exon 4
EZH2	entire gene	*SF3B1*	exon 13-16
FBXW7	exon 9-11	*SMC1A*	exon 2, 11, 16 and 17
GATA1	exon 2	*SMC3*	exon 10,13,19,23,25 and 28
GATA2	exon 2-6	*SRSF2*	exon 1
GNAS	exon 8-9	*STAG2*	entire gene except 7 and 17
HRAS	exon 2-3	*TET2*	exon 3-11,
IDH1	exon 4	*TP53*	exon 2-10
IDH2	exon 4	*U2AF1*	exon 2 and 6
IKZF1	entire gene	*WT1*	exon 7 and 9
JAK2	exon 12 and 14	*ZRSR2*	exon 2-11
KDM6A	entire gene		

Supplementary Table 3. Summary of identified CHIP variants with the TruSight panel.

Gene	Chr.	Position	Reference	Alternative	Consequence	VAF	Amino_acid_change
DNMT3A	2	25457242	C	T	missense_variant	0,0711	R/H
DNMT3A	2	25463265	G	C	missense_variant	0,0669	P/R
DNMT3A	2	25463586	C	A	missense_variant	0,0462	G/V
DNMT3A	2	25469948	T	C	missense_variant	0,064	Y/C
SF3B1	2	198266542	T	C	missense_variant	0,046	Y/C
SF3B1	2	198267306	C	T	missense_variant	0,067	R/K
GATA2	3	128199938	G	A	missense_variant	0,4648	P/L
KIT	4	55561758	G	T	missense_variant	0,5082	V/L
TET2	4	106155620	C	A	missense_variant	0,5236	P/H
TET2	4	106155620	C	A	missense_variant	0,5538	P/H
TET2	4	106157485	G	A	missense_variant	0,5097	E/K
TET2	4	106182953	C	-	frameshift_variant	0,0426	T/X
TET2	4	106196424	C	G	stop_gained	0,19	S/*
FBXW7	4	153245527	A	T	missense_variant	0,0559	V/E
TP53	17	7577120	C	T	missense_variant	0,0492	R/H
ASXL1	20	31022752	C	T	missense_variant	0,4663	A/V
ZRSR2	X	15827389	C	T	stop_gained	0,5025	R/*
ZRSR2	X	15838431	C	T	missense_variant	0,4038	A/V
ZRSR2	X	15840941	C	G	missense_variant	0,9984	A/G
BCOR	X	39923718	G	C	missense_variant	0,0569	P/A
BCOR	X	39923722	G	C	missense_variant	0,0574	D/E
BCOR	X	39923726	G	C	missense_variant	0,0566	S/W
BCOR	X	39923727	A	T	missense_variant	0,0589	S/T
BCOR	X	39923732	A	C	missense_variant	0,0545	V/G
KDM6A	X	44922890	C	T	missense_variant	0,9953	T/M
BCORL1	X	129148792	T	C	missense_variant	0,0577	S/P

Supplementary references:

1. Dumanski JP, Halvardson J, Davies H, Rychlicka-Buniowska E, Mattisson J, Moghadam BT, et al. Immune cells lacking Y chromosome show dysregulation of autosomal gene expression. Cell Mol Life Sci. 2021.
2. Forsberg LA, Rasi C, Malmqvist N, Davies H, Pasupulati S, Pakalapati G, et al. Mosaic loss of chromosome Y in peripheral blood is associated with shorter survival and higher risk of cancer. Nat Genet. 2014;46(6):624-8.
3. Danielsson M, Halvardson J, Davies H, Torabi Moghadam B, Mattisson J, Rychlicka-Buniowska E, et al. Longitudinal changes in the frequency of mosaic chromosome Y loss in peripheral blood cells of aging men varies profoundly between individuals. Eur J Hum Genet. 2020;28(3):349-57.
